# Transmembrane Activator and CAML Interactor (TACI): Another Potential Target for Immunotherapy of Multiple Myeloma?

**DOI:** 10.3390/cancers12041045

**Published:** 2020-04-23

**Authors:** Shengli Xu, Kong-Peng Lam

**Affiliations:** 1 Bioprocessing Technology Institute, Agency for Science, Technology and Research, Singapore 138668, Singapore; 2 Department of Physiology, Yong Loo Lin School of Medicine, National University of Singapore, Singapore 117593, Singapore; 3 Department of Microbiology and Immunology, Yong Loo Lin School of Medicine, National University of Singapore, Singapore 117545, Singapore; 4 School of Biological Sciences, Nanyang Technological University, 60 Nanyang Drive, Singapore 637551, Singapore

**Keywords:** TACI, plasma cell, multiple myeloma, immunotherapy

## Abstract

Multiple myeloma (MM) has emerged as the next most likely oncological or hematological disease indication amenable for cellular immunotherapy. Much of the attention has been focused on B cell maturation antigen (BCMA) as a unique cell surface protein on myeloma cells that is available for monoclonal antibodies, antibody drug conjugates (ADCs), T-cell redirecting bispecific molecules, and chimeric antigen receptor (CAR) T cell targeting. BCMA is a member of the tumor necrosis factor receptor (TNFR) superfamily that binds two ligands B-cell activating factor (BAFF) and a proliferation-inducing ligand (APRIL) and mediates the growth and survival of plasma and MM cells. Interestingly, transmembrane activator and CAML interactor (TACI), another TNFR superfamily member, also binds the same ligands and plays largely overlapping roles as BCMA in normal plasma and malignant MM cells. In this article, we review the biology of TACI, focusing on its role in normal B and plasma cells and malignant MM cells, and also discuss various ways to incorporate TACI as a potential target for immunotherapies against MM.

## 1. Introduction

Multiple myeloma (MM) is a B-cell neoplasia characterized by the uncontrolled growth and accumulation of malignant plasma cells in the bone marrow (BM). Although the past two decades have seen considerable advances in the treatment of MM with the introduction of immunomodulatory drugs, proteasome inhibitors, and monoclonal antibodies such as Elotuzumab and Daratumumab that target signaling lymphocytic activation molecule F7 (SLAMF7) and CD38, respectively [1], MM still remains incurable with most patients eventually succumbing to the disease [2,3]. Meanwhile, a large fraction of MM patients are resistant to current therapies due to the fact that MM cells have high propensity for clonal heterogeneity and have complex interactions with the BM environment, which hinders disease treatment [4,5]. Therefore, new effective therapies are needed for the treatment of relapsed/refractory MM.

**Figure 1:**
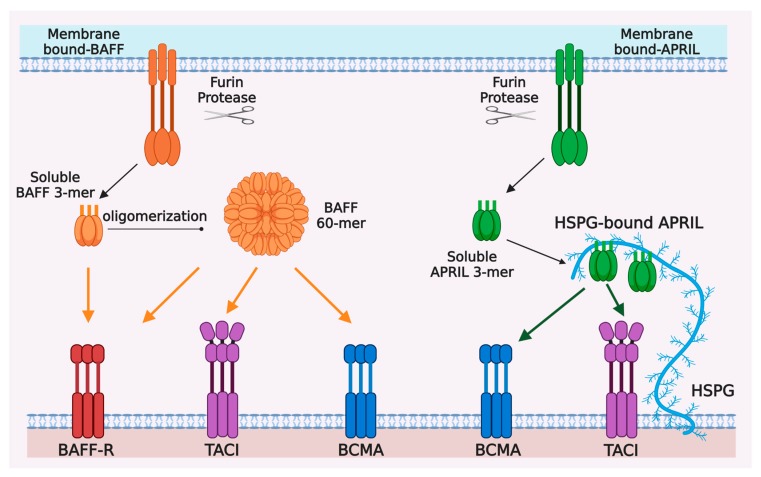
Ligand-receptor interactions in the BAFF/APRIL and BAFF-R/BCMA/TACI system. BAFF and APRIL are first synthetized as type II transmembrane proteins, mainly in myeloid and stromal cells, and processed by furin protease into trimeric soluble cytokines BAFF 3-mer and APRIL 3-mer. Twenty soluble BAFF 3-mers can be further oligomerized into a virus-like particle, called BAFF 60-mer. BAFF-R, BCMA, and TACI are type I transmembrane proteins belonging to tumor necrosis factor receptor (TNFR) superfamily and mainly expressed by B cells at different stages of differentiation. BAFF-R and BCMA have only one cysteine-rich domains (CRD) extracellularly, whereas TACI has two CRDs for ligand-binding. BAFF-R only binds BAFF, which can be BAFF 3-mer, BAFF 60-mer or membrane-bound BAFF. BCMA can bind both BAFF and APRIL and has higher binding affinity with APRIL than that with BAFF. TACI also binds to BAFF and APRIL, but it seems to respond better to oligomeric ligands, i.e., BAFF 60-mer or HSPG-bound APRIL, than trimeric soluble ligands.

The BM microenvironment is known to be critical for the growth and survival of myeloma cells by protecting them from spontaneous and drug-induced apoptosis [6,7,8,9]. The interaction between myeloma and BM stromal cells is mainly mediated by soluble factors and adhesion molecules. B-cell activating factor (BAFF) and a proliferation-inducing ligand (APRIL) are two important cytokines that have been shown to play critical roles in MM growth and survival [10,11,12,13]. BAFF, which is also known as TALL-1, THANK, BLyS, and zTNF4, was first identified as a member of the tumor necrosis factor (TNF) ligand superfamily that can stimulate B cells [14,15,16,17,18]. APRIL was first cloned as a TNF ligand that can stimulate tumor cell proliferation [19,20]. Both BAFF and APRIL bind two receptors called BCMA (B cell maturation antigen) and TACI (transmembrane activator and CAML interactor) [15,21,22,23,24,25,26]. BAFF also binds another receptor called BAFF-R or BR3 [27,28]. APRIL does not bind BAFF-R but binds heparan sulphate proteoglycan (HSPG) in both the extracellular matrix and on the surface of cells like plasma cells, triggering APRIL multimerization [29,30] (Figure 1). Consistent with the important roles of BAFF and APRIL in MM, BCMA, and TACI were found to be expressed on MM cells and mediate the signals induced by BAFF and APRIL to support MM growth and survival [10,11,12].

The important roles of BAFF/APRIL and their receptors BCMA/TACI in MM make them potential therapeutic targets for the development of new treatment for relapsed/refractory MM. In recent years, a number of therapeutic modalities targeting BCMA, including antibody-drug conjugates (ADCs), T-cell redirecting bispecific molecules or antibodies and chimeric antigen receptor (CAR)-T cells have been developed and shown to demonstrate promising therapeutic effects preclinically and clinically [31,32,33]. However, some challenges, such as variable and often low level of BCMA expression on MM cells and antigen-negative tumor escape after initial BCMA-targeted immunotherapy, result in unsustained remission [31]. Thus, there is a need to explore other targets in this subgroup of ligands and receptors for the development of new modalities that can supplement or improve BCMA-targeted immunotherapy. In this review, we will summarize the role of TACI in normal B cell physiology and MM pathophysiology and the progress made in immunotherapeutic treatments against MM that incorporate TACI-targeting in the strategy.

## 2. The Role of TACI in Normal B Cells and MM

### 2.1. TACI is Important for Regulating B Cell Functions

The transmembrane activator and CAML interactor (TACI) is a member of the tumor necrosis factor receptor (TNFR) superfamily and designated as TNFRSF13B. It was first identified as a binding partner of calcium-modulator and cyclophilin ligand (CAML) protein in Jurkat T cells and functioned as a co-inducer of nuclear factor of activated T cells (NF-AT) transcription factor. Cross-linking of TACI by anti-TACI antibodies also led to the activation of transcription factors AP-1 and NF-κB [34]. Later on, together with BCMA, TACI was identified as a receptor that can bind and transduce the B cell-activating signals of BAFF and APRIL [15,22,23,24,25,35], and has been shown to play important roles in B cell activation and differentiation (Table 1).

Although BAFF and APRIL are known to play stimulatory roles in B cells, the initial characterization of TACI-deficient mice showed that TACI played an unexpected inhibitory role in B cell activation in vivo, as TACI−/− mice manifested splenomegaly with increased mature B cells [36,37]. On the other hand, BCMA−/− mice exhibited normal B cell development and activation [52]. The phenotypes of TACI−/− and BCMA−/− mice were in great contrast to that of BAFF−/− and TACI-Ig transgenic mice, which displayed severe B cell developmental block at the transitional B cell stage and had dramatically reduced mature B cell numbers [38,53]. This discrepancy led to the speculation that there could be other receptors responsible for BAFF’s stimulatory function in B cells. The paradox was resolved by the later discovery of BAFF-R, a third receptor for BAFF [27,28]. Importantly, A/WySnJ mice, with natural mutation in the BAFF-R locus, and BAFF-R knockout mice both had drastically decreased mature B cell population [27,54], a phenotype qualitatively similar to that of BAFF−/− mice [53]. These results suggest that BAFF-R is the principal receptor for BAFF-mediated maturation and survival of B cells.

**Figure 2:**
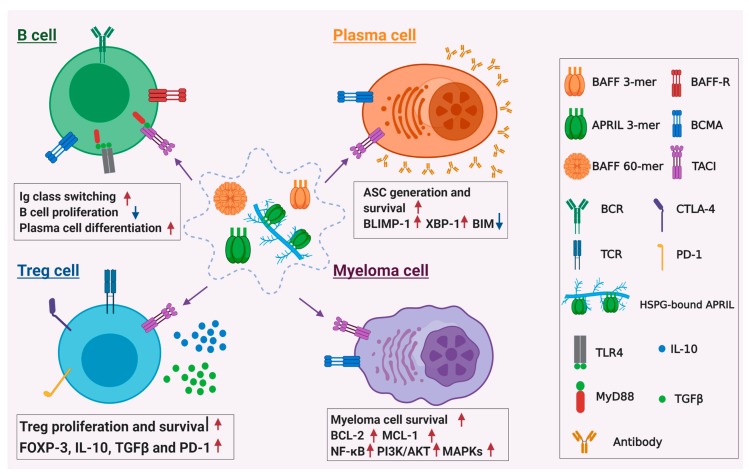
Role of TACI in B cell physiology and MM pathophysiology. For normal B cells, TACI regulate immunoglobulin class switching upon engagement by BAFF or APRIL. It transduces the activation signals via interacting with adaptor protein MyD88 and cooperates with signaling through TLRs, such as TLR4, to promote immunoglobulin class switching. TACI can also regulate plasma cell differentiation and survival by upregulating transcriptional factors Blimp-1 and XBP-1 and downregulating pro-apoptotic protein Bim. For pathogenesis of MM, TACI mediates the signals of BAFF and APRIL to activate multiple downstream signaling pathways, including NF-κB, PI3k/Akt, and MAPKs pathways, leading to upregulation of anti-apoptotic proteins BCL-2 and MCL-1, which enhance MM cell survival. TACI-mediated signaling can also support immunosuppressive tumor microenvironment in the bone marrow of MM patients by promoting the survival of regulatory T cells and their inhibitory functions.

Although TACI is dispensable for B cell maturation and survival, it was found to be important for BAFF- and APRIL-induced immunoglobulin (Ig) isotype switching to IgG1, IgA, and IgE in murine B cells [39] (Figure 2). It was further demonstrated that the adaptor protein MyD88 was recruited to the cytoplasmic tail of TACI upon its engagement by BAFF and APRIL [46]. The association of TACI and MyD88 triggers NF-κB activation, Ig germline gene transcription, and activation-induced cytidine deaminase expression. Interestingly, mutations in the *TACI* gene were found in about 10% of patients with common variable immune deficiency (CVID), a disease that manifests with hypogammaglobulinemia, defective antibody production, recurrent infections, and autoimmunity [40,41]. These patients were typically found to have a heterozygous *TACI* C104R mutation that abolishes ligand binding and results in the failure of B cells to produce class-switched antibodies [55,56,57]. Paradoxically, CVID patients with a single *TACI* mutation are also prone to autoimmune cytopenias, whereas patients devoid of functional TACI are protected from autoimmunity [58]. This apparent discrepancy was reconciled by a study showing that Toll-like receptor (TLR)-7 and 9-mediated signaling pathways were severely impaired by the complete loss of function of TACI, which was likely to be protective against autoimmunity developing from TACI-deficient autoreactive naive B cells [44].

### 2.2. TACI is Important for the Differentiation and Survival of Plasma Cells

In addition to its role in Ig class switching, TACI is also found to be essential for the differentiation and survival of plasmablasts and plasma cells (Figure 2). When murine B cells were cultured with agonistic anti-CD40 antibody and IL-4, the concurrent engagement of their TACI receptor with anti-TACI antibody significantly led to an increase in the fraction of CD138^+^ cells, suggesting that TACI-mediated signaling promotes CD40-stimulated B cells to differentiate into plasmablasts [42]. TACI was also shown to be important for LPS-induced plasmablasts formation. In wild-type (WT) B cells, APRIL can strongly synergize with sub-optimal doses of LPS to drive the differentiation program of plasma cells, as evidenced by the elevated expression levels of CD138, B lymphocyte induced maturation protein-1 (Blimp-1), interferon regulatory factor-4 (IRF-4), and the spliced form of X-box binding protein-1 (XBP-1) and enhanced antibody secretion [45]. The synergistic effect of APRIL is mainly dependent on TACI, as TACI−/− but not BCMA−/− B cells had impaired IgM, IgA, IgG1, and IgE secretion. Furthermore, the in vivo antibody responses to suboptimal dose of T cell-independent type I antigen, 2,4,6-Trinitrophenol (TNP)-LPS was also defective in TACI−/− mice compared with WT animals. Another study demonstrated that TACI was equally important for the in vitro survival of plasmablasts differentiated in vivo. Treatment with BAFF 60-mer or cross-linked APRIL could significantly improve the in vitro survival of plasmablasts isolated from the spleens of mice immunized with tetanus toxoid [59]. BAFF 60-mer- or cross-linked APRIL can increase the number of antibody secreting cells by 6- to 10-fold but the effect was impaired by TACI-deficiency and to a lesser extent, by BCMA-deficiency, and was completely abrogated by the combined deficiencies of TACI and BCMA. On the other hand, BAFF 3-mer, which can only engage BAFF-R exhibited very marginal effect on the survival of plasmablasts. These data together suggest that the in vitro survival of BAFF and APRIL on plasmablast is mainly mediated by TACI and to a lesser extent, BCMA, whereas BAFF-R is probably not required for plasmablast survival.

Studies of TACI-/- mice further revealed that TACI is also important for plasma cell differentiation and survival in vivo. TACI was demonstrated to be important for the in vivo differentiation of plasma cells in response to T cell-independent type II antigen, NP-Ficoll [43]. TACI-deficient mice generated lesser amount of NP-specific antibody secreting cells (ASCs) compared to WT mice. In addition, TACI-deficient B cells were found to remain in cell cycle longer than WT cells and have impaired plasma cell differentiation in response to NP-Ficoll, suggesting that TACI signaling attenuates B cell proliferation to allow for plasma cell differentiation. When challenged with proteinous antigens, TACI-deficient mice also manifested defective T cell-dependent antibody response [47,48]. Upon immunization, TACI-deficient mice produced a smaller amount of antigen-specific antibodies due to defective ASC generation. Although TACI was dispensable for Blimp-1 expression, it was important to sustain Blimp-1 expression and long-lived plasma cell differentiation [47]. Furthermore, TACI was also shown to be required for APRIL-mediated downregulation of the pro-apoptotic gene *Bim*, and deletion of *Bim* can partially rescue the defect in antigen-specific ASC production in TACI-deficient mice [48].

Interestingly, unlike the murine *TACI* gene, human *TACI* gene undergoes alternative mRNA splicing and produces two isoforms―short and long isoforms, which has one or two ligand-binding domains, respectively. The short isoform was predominantly found in CD27^+^, TLR9-activated peripheral B and marginal zone B cells [49]. When the short TACI isoform was overexpressed in human pre-B cells, the cells displayed enhanced NF-κB activation, MyD88 colocalization and upregulation of *Blimp-1* and *Xbp-1* expression. These TACI-overexpressing cells also lost the cell surface expression of CD19 and IgG and became CD138^+^ and acquired the morphology of plasma cells. In contrast, B cells overexpressing the long TACI isoform had significantly less expression of *Blimp-1* and *Xbp-1* and retained cell surface CD19 and IgG expression and remained CD138 negative. Although the detailed mechanism underlying the regulation of *TACI* alternative splicing in human B cells remains unknown, these results suggest that the short isoform might control plasma cell differentiation in humans.

### 2.3. TACI in the Pathophysiology of MM

MM is thought to be a progressive disease arising from multiple genetic lesions to plasma cells, which ultimately result in malignant myeloma cells with uncontrolled proliferative potential accumulating in the BM. Although various genetic alterations initiate plasma cell neoplasia, the survival and proliferation of myeloma cells still largely depend upon the BM microenvironment where they are in close contact with stromal cells. Several autocrine or paracrine soluble factors are known to be critical for promoting the survival and growth of myeloma cells and these include interleukin 6, interferon alpha, insulin-like growth factor-1, hepatocyte growth factor, and heparin-binding epidermal growth factor-like growth factor [60,61,62,63,64]. In addition to their important roles in normal B cell homeostasis, BAFF and APRIL are also found to be involved in the pathogenesis of MM [10,11,12,13]. It was shown that BAFF and APRIL are expressed in cells from BM microenvironment and are important for the growth and survival of MM cells (Figure 2). They can activate multiple signaling pathways including NF-κB, phosphatidylinositol-3 (PI-3) kinase/AKT and mitogen-activated protein kinase (MAPK) pathways in myeloma cells and upregulate their expression of anti-apoptotic proteins such as MCL-1 and BCL-2. Similar to the situation found in normal plasma cells, the pro-survival and proliferative function of BAFF and APRIL are mainly mediated by BCMA and TACI in MM [10,12].

In contrast to the near ubiquitous expression of BCMA in almost all human MM cell lines (HMCLs) and MM patients’ primary myeloma cells studied so far, the expression of TACI was found to be more variable in MM cells. When first examined by RT-PCR analysis, TACI mRNA was detected in 8 of 13 HMCLs studied and its expression level seemed to be higher than that of BAFF-R but lower than that of BCMA [10]. Similarly, when the expression of these receptors was investigated in CD138^+^ myeloma cells isolated from a larger cohort of 36 MM patients by microarray analysis, TACI was again detected to be expressed at a higher level than that of BAFF-R [12]. In another study, the surface expression of TACI was also examined by flow cytometry in HMCLs and primary MM cells. It was found that 5 out of 6 HMCLs were TACI positive and 3 out of 3 patients’ CD138^+^ MM cells also expressed TACI on their cell surface with 2 primary samples expressing a quite high TACI level [11].

A more detailed characterization of 17 HMCLs using microarray analysis further suggested that gene expression of *TACI* rather than *BCMA* yielded a functional BAFF-binding receptor [64]. This study showed that only HMCLs that expressed *TACI* could bind BAFF. HMCLs having no detectable expression of *TACI* could not bind to BAFF even though they had high expression levels of *BCMA*. Similar results were also obtained when purified primary myeloma cells were examined. Myeloma cells that highly expressed *BCMA* but weakly expressed *TACI* based on RT-PCR analysis, failed to bind BAFF. In contrast, primary myeloma cells that highly expressed *TACI* could bind BAFF [64]. However, in this study, the expression of TACI and BCMA was only assessed by microarray or RT-PCR analysis and the surface expression of these two receptors was not examined. In addition, it was not studied if these cells could bind APRIL, which is another important factor for myeloma cell survival.

The same study also revealed that myeloma cells with a high level of TACI (TACI^high(hi)^) had higher expression of genes encoding for intercellular communication signaling, cytoskeleton-associated proteins, and intracellular signal transduction. In contrast, TACI^low(lo)^ myeloma cells overexpressed genes coding for proteins involved in cell cycle and nuclear functions [64]. Interestingly, when compared to the gene expression profiles of normal plasma cells and plasmablasts in the BM, it was found that TACI^hi^ myeloma cells exhibited a gene signature resembling that of mature BM plasma cells with higher expression of genes encoding for proteins involved in intercellular communication and signal transduction. On the other hand, TACI^lo^ myeloma cells displayed a gene profile similar to that of plasmablasts with a higher expression of cell cycle genes. The gene signatures of TACI^hi^ and TACI^lo^ myeloma cells might reflect the degree of dependency of the growth of these two groups of myeloma cells on the BM microenvironment. The TACI^hi^ myeloma cells with the mature plasma cell gene signature could depend more on the BM environment. On the other hand, TACI^lo^ myeloma cells with the gene signature of plasmablasts could have an attenuated dependence on the BM environment. Interestingly, some clinical differences were also found between patients with TACI^lo^ or TACI^hi^ phenotypes. For example, TACI^lo^ patients had clinical parameters associated with poor prognosis, such as an increase in the percentage of stage III MM, a decrease in hemoglobin level, and an increase in the percentage of bone lesions, suggesting that TACI expression level might be an independent prognostic factor for MM progression or disease severity.

In addition to being important for the growth and survival of myeloma cells, TACI has also been shown to impact the immunosuppressive microenvironment in the BM of MM patients. Recently, a study showed that TACI can transduce signals in regulatory T (Treg) cells upon the engagement of APRIL, contributing to the immunosuppressive microenvironment of MM [51]. It was reported that TACI expression was significantly higher in Tregs than conventional T cells from the same MM patients. Treatment with APRIL could promote the proliferation and survival of Tregs and augment the expression of Foxp3, IL-10, TGFβ1, and PD-L1 in Tregs (Figure 2), and these effects could be inhibited by anti-APRIL neutralizing antibody. Interestingly, the study also showed that both the number of regulatory B (Breg) cells from the bone marrow of MM patients and IL-10 production by these Breg cells were increased by TACI-mediated APRIL signaling. This is in line with results from a previous study that showed that BAFF could also mediate IL-10 production by Breg cells via TACI in healthy donors and chronic lymphocytic leukemia patients [50]. These data together suggest that TACI-mediated signaling of APRIL, and probably also BAFF, could contribute to the immunosuppressive tumor microenvironment found in the BM of MM patients, which could potentially allow MM cells to escape from anti-tumor immune surveillance.

## 3. Targeting TACI in Immunotherapy Against MM

The significance of BAFF and APRIL, and their receptors BCMA and TACI, in the growth and survival of myeloma cells suggests that these paired ligands and receptors can be targets for the development of new therapies against MM. Indeed, a number of BCMA-targeting immunotherapies, including anti-BCMA antibody-drug conjugate [65,66,67,68], anti-BCMA bi-specific T cell engager (BiTE) [69,70], and BCMA-targeting chimeric antigen receptor T cells [71,72,73,74,75,76], have been developed and are at different stages of preclinical and clinical development for the treatment of resistant/relapsed MM and they have yielded promising results. However, the variable density of BCMA on MM cells in different patients and the propensity of target escape after initial BCMA-targeting immunotherapy may compromise therapeutic efficacy. For example, BCMA on the cell surface can be cleaved by γ-secretase [77], and the resulting reduced cell surface level of BCMA and increased serum level of soluble BCMA could potentially limit the efficacy of BCMA-directed adoptive T-cell therapy by inhibiting CAR T-cell recognition of BCMA on myeloma cells [33,78,79]. Therefore, targeting other members in this ligand-receptor subgroup such as TACI, either alone or in combination with BCMA-targeting therapies, might achieve better clinical efficacy or improve the treatment outcome for MM patients. Here, we summarize some preclinical progress in the development of TACI-based immunotherapies for the treatment of MM.

### 3.1. Soluble TACI-Fc Fusion Protein Inhibits Myeloma Cell Growth and Survival

TACI-Fc fusion protein, which can neutralize BAFF and APRIL, was first shown to be able to inhibit the autonomous proliferation of HMCLs RPMI8226 and L363 [10]. It was also found that TACI-Fc could significantly induce the apoptosis of primary myeloma cells cultured with components of the BM microenvironment and recombinant IL-6. In experiments involving co-cultures of HMCL RPMI8226 or CD138^+^ myeloma cells with osteoclasts, the addition of TACI-Fc led to substantially enhanced apoptosis of MM cells [13]. Furthermore, TACI-Fc can potentiate the inhibitory effect of dexamethasone or anti-IL-6 antibody on the survival of myeloma cells [10]. In addition, TACI-Fc treatment could reduce dendritic cell-supported clonogenic potential of HMCL U266 and primary myeloma cells [80].

The in vivo effect of TACI-Fc on primary myeloma growth was further investigated using primary myelomatous SCID-hu mice [81]. When myelomatous SCID-hu mice with patient myeloma cells engrafted were treated with TACI-Fc, ~80% of the mice had reduced myeloma burden or decreased myeloma growth rate. Interestingly, when the response of SCID-hu mice to TACI-Fc treatment was correlated with TACI expression levels in the myeloma cells, it was found that the response to TACI-Fc was superior in TACI^high^ myeloma cells compared with TACI^low^ cells. It was also shown that treatment with BAFF-R-Fc did not have any significant inhibitory effect on myeloma cell growth, implying that blockade of APRIL is more beneficial than the blockade of BAFF for MM patients. These studies suggest that targeting APRIL is an alternative therapeutic strategy, especially for patients with TACI^high^-expressing myeloma cells.

### 3.2. Antagonistic Anti-APRIL Antibody Inhibits MM Growth and Modulates Immunosuppressive BM Microenvironment

Using antagonistic antibody to neutralize APRIL is another effective strategy to abrogate the pro-survival and growth-promoting signaling pathways mediated by TACI and BCMA. It was shown that the binding of APRIL to TACI or BCMA can be efficiently blocked by an antagonistic anti-human APRIL monoclonal antibody hAPRIL.01A (01A) [82]. In vitro studies showed that the 01A antibody can inhibit APRIL-induced survival of HMCLs and it exerted its cytotoxicity effect even in the presence of protective BM myeloid cells such as osteoclasts, macrophages, and plasmacytoid dendritic cells [83]. The 01A antibody can reduce APRIL-induced MM cell adhesion and migration through blocking NF-κB signaling pathway. In the SCID-hu mouse model, 01A antibody was found to suppress MM growth in the BM microenvironment in vivo [83].

Recently, the inhibitory effect of the antagonistic anti-APRIL antibody on BM microenvironment was also evaluated. It was reported that TACI-dependent survival and proliferation of Treg cells as well as their upregulation of immunosuppressive factors, such as IL-10, TGFβ1, and PD-L1 were all abrogated by treatment with the antagonistic anti-APRIL antibody [51]. These results suggest that antagonistic anti-APRIL antibody can inhibit MM by targeting both myeloma cells and the immunosuppressive BM microenvironment, on which myeloma cells rely on for survival.

### 3.3. Incorporating TACI in CAR-T Cells Targeting of MM

CAR-T cell therapy is an innovative adoptive cell therapy that has revolutionized the treatment landscape of certain hematological malignancies. Recently, the US FDA approved two CD19-targeted CAR-T cell therapies, Kymriah and Yescarta, for the treatment of B-cell acute lymphoblastic leukemia (ALL) and diffuse large B-cell lymphoma (DLBCL). The treatment is currently also being investigated for other B cell malignancies, including MM. In addition, several BCMA-targeting CAR-T cell immunotherapies have shown promising results in the early-phases of clinical trials [71,72,73,74,75,76].

Although BCMA is a promising target, challenges such as its low level expression in some patients and antigen-negative tumor escape due to BCMA downregulation or shedding may compromise the clinical efficacy of BCMA-targeted CAR-T cell therapy. Also, relatively high T-cell doses were needed to achieve durable remissions [71]. It was hypothesized that dual-antigen targeting would increase the level of targetable antigen on MM cells and reduce the incidence of antigen-negative tumor escape for achieving better therapeutic efficacy and long-term disease control. A recent study showed that a CAR design using the receptor-binding domain of APRIL as the tumor antigen-targeting domain (APRIL-based chimeric antigen receptors; ACAR), which recognizes both BCMA and TACI on MM cells, could potentially improve clinical outcomes [84]. In that study, it was shown that about 78% of MM patients examined co-expressed BCMA and TACI on the surface of their MM cells, although the abundance of TACI was 3- to 4-fold lower than that of BCMA. It was demonstrated that ACAR-T cells could cause target cytolysis at low densities of BCMA and TACI antigen and at a low effector to target cell ratio, even in the presence of soluble APRIL, BCMA, and TACI. In addition, the killing of BCMA^+^TACI^+^ cells by ACAR-T cells was not affected by an anti-BCMA blocking antibody, indicating that ACAR-T cells could maintain MM-killing efficacy possibly by targeting TACI on MM cells even when BCMA is downregulated. In vivo study further showed that when compared to CAR T cells targeting BCMA alone, ACAR-T cells could achieve continuous in vivo tumor suppression even in the event of BCMA downregulation [84] Thus, ACAR-T cells could represent an alternative strategy for the treatment of MM patients co-expressing BCMA and TACI but with BCMA expression downregulated or lost. Although it was suggested that lower expression of TACI, as determined by mRNA expression, in MM cells correlated with poorer prognosis of the patients [64], the cell surface density of TACI did not correlate with the killing effect of ACAR-T cells. These cells were efficacious even at low effector to target cell ratio and in the presence of anti-BCMA blocking antibody [84], arguing that lower cell surface level of TACI might not necessarily correlate with less efficacious therapeutic effect of TACI-targeting immunotherapy.

In another recent study using APRIL-based, 2nd generation CAR that targets BCMA and TACI simultaneously, it was further shown that APRIL in a trimeric format (Tr-iAPRIL) as the antigen-binding domain, displayed enhanced binding to BCMA and TACI and exhibited greater target cytolysis activity against MM compared to monomeric APRIL CAR [85]. This study also demonstrated that the Tri-APRIL CAR-T cells could achieve long-term proliferation and acquire better polyfunctionality than the monomeric APRIL CAR-T cells in a xenograft mouse model. These studies further suggest that dual-specific and trimeric APRIL-based CAR is a promising therapeutic modality for the treatment of MM.

## 4. Conclusions and Perspectives

Immunotherapy against MM is gaining momentum as more therapeutic modalities are being developed and tested in preclinical and clinical settings. A considerable number of therapeutic modalities that are being developed have focused on targeting the BCMA protein expressed on MM cells. These BCMA-targeted therapies, such as the ADC GSK2857916 [65,66,67], bispecific antibody AMG 420 [69], and several CAR T-cell therapeutic agents including bb2121 [73], NIH CARBCMA [71,72], and LCAR-B38M [74] have demonstrated promising preliminary clinical results. However, challenges such as variable and often low expression of BCMA in some MM patients and antigen-negative tumor escape due to downregulation or shedding of BCMA could compromise the efficacy of these therapeutic agents, indicating that there is room for scientists and clinicians to continue to develop other innovative therapies. Being a receptor that is expressed on MM cells and has largely overlapping functions with BCMA, TACI offers promise as an additional cell surface target for the development of new therapies that can supplement or improve upon BCMA-targeted therapies. Examples are TACI-Fc that can neutralize BAFF and APRIL and anti-APRIL neutralizing antibody, both of which can abolish the signaling through BCMA and TACI, and the more recently reported monomeric and trimeric APRIL-based CAR-T cells that recognize BCMA and TACI on MM cells simultaneously. These therapeutic agents have already shown some efficacies in preclinical studies. Additional strategies would include developing different therapeutic modalities that directly target TACI on MM cells, including TACI-targeted ADCs and bispecific antibodies as well as TACI-based CAR-T or CAR-NK cells.

## Figures and Tables

**Table 1:** Studies for the role of TACI in B cell function and differentiation.

Year	Major Findings	Reference
1997	TACI was cloned as a calcium-modulator and cyclophilin ligand (CAML) associated protein belonging to tumor necrosis factor receptor superfamily. Engagement of TACI led to activation of transcription factors AP-1, NF-AT, and NF-κB.	[34]
2000	TACI was identified as a receptor that binds BAFF and APRIL.	[15,22,23,24,25,35]
2001	TACI-deficient mice exhibited increased B cells and splenomegaly, whereas TACI-Ig transgenic mice had defective B cell development and reduced circulating immunoglobulins.	[36,37,38]
2005	TACI is important for immunoglobulin class switching in murine B cells. Mutations of TACI are associated with common variable immunodeficiency in humans	[39,40,41]
2007	Engagement of TACI promotes plasmablast differentiation in vitro. TACI-deficient B cells had defective plasma cell differentiation in response to T cell-independent type 2 antigens	[42,43]
2008	TACI is solely activated by oligomeric BAFF and APRIL and required for the survival of plasmblasts.	[44]
2009	TACI drives plasma cell differentiation in LPS-activated B cells and is required for T cell-independent type 1 antibody response.	[45]
2010	TACI signals via associating with adaptor MyD88 to trigger immunoglobulin class switching.	[46]
2011	TACI is important for differentiation of long-lived antibody-secreting cells by promoting sustained Blimp-1 expression.	[47]
2012	TACI promotes the survival of long-lived antibody-secreting cells by mediating APRIL-induced downregulation of pro-apoptotic gene *Bim.*	[48]
2015	Overexpression of short isoform of TACI in human pre-B cells promotes their differentiation into plasma cells.	[49]
2017	BAFF-mediated IL-10 production by Breg cells via TACI in healthy donors and CLL patients	[50]
2019	APRIL increases number of IL-10-producing Breg cells and their IL-10 production via TACI expressed on Breg cells from the bone marrow of MM patients.	[51]

TACI, transmembrane activator and calcium modulator and cyclophilin ligand interactor; AP-1, activation protein 1; NF-AT; nuclear factor of activated T cells; NF-κB, nuclear factor kappa-light-chain-enhancer of activated B cells; BAFF, B cell activating factor; APRIL, a proliferation-inducing ligand; LPS, lipopolysaccharides; MyD88, myeloid differentiation primary response protein 88; Blimp-1, B lymphocyte-induced maturation protein-1, Bim, BCL2-interacting mediator of cell death, CLL, Chronic Lymphocytic Leukemia.
